# Oxford Textbook of Medicine

**Published:** 1987-11

**Authors:** J. D. Davies

**Affiliations:** University of Bristol


### Book Review

**OXFORD TEXTBOOK OF MEDICINE**
**Eds. D. J. Weatherall, J. G. G. Ledingham and D. A. Warrell**
**Oxford University Press, Oxford, 1987**
**pp. approx 2500, hardback.**
**Second edition, 2 volumes**
**ISBN 0 19 261551 3 (set).**
**Price £95.00**

*Beside, he was a shrewd philosopher,*
*And had read ev'ry text and gloss over.*
Samuel Butler, *Hudibras*, 1663–1678

I scarcely know how to begin this review. The *Oxford Textbook of Medicine* is no ordinary book. For a start it weighs about one and a quarter stone, and as I know to my cost it is not easy to carry home on foot. An African-style head cushion might help you here. Secondly, as a well meaning colleague said unexpectedly tartly – well you've got a lot of reading to do before filing your review. True, but I've enjoyed the job. How the 17th century editors of King James' English Authorised and Bishop Morgan's Welsh versions of the Bible coped without word-processors I'll never know.

We all qualify, get embroiled in our own particular lines and can easily lose touch with other fields. This massive book offers considerable help in medicine, by which I do not mean only the American sort of 'internal medicine'. This is Medicine. Amazingly, despite the roll call of over 350 individual authors the general style is pleasing and comparatively uniform. Where called for, there are many clear and useful illustrations.

The *Oxford Textbook of Medicine* is undoubtedly *the* bible of modern medicine. No, not just the high-tech type. Both the mundane and the abstruse topics are considered. There is everything for undergraduates, membership candidates, practitioners in third-world conditions, researchers and even mere pathologists. As the computer magazines say about lesser offerings, it is all wrapped up in an exceptionally attractive bundle. Apart from the general small revisions, with new and extensively rewritten chapters, this second edition is also considerably improved by now having a full index at the end of both volumes.

I personally am lost in admiration of this production. Expensive it may seem to be at first sight. Would that the two main Defence Unions were to give such value for money! However I can't think of any better way to reduce medical litigation, or to give an enjoyable and informative medical read than the second edition of this exceptional book.

J. D. Davies

University of Bristol

